# A 3-year prospective study to assess clinical characteristics and risk factors for exacerbations in patients with asthma-COPD overlap based on the GINA guideline compared with patients with asthma and COPD.

**DOI:** 10.1186/s12931-026-03643-0

**Published:** 2026-03-29

**Authors:** Fanny Wai San KO, Ken Ka Pang CHAN, Timothy Chi Chun NG, Rachel Lai Ping LO, Jenny Chun Li NGAI, Kin Wang TO, Wing Ho YIP, Joyce Ka Ching NG, Christopher CHAN, Charlotte Ho Ying LAU, David Shu Cheong HUI

**Affiliations:** 1 Department of Medicine & Therapeutics, Faculty of Medicine, The Chinese University of Hong Kong, Hong Kong, China; 2 Li Ka Shing Institute of Health Science, Faculty of Medicine, Chinese University of Hong Kong, Hong Kong, China; 3 Department of Medicine & Therapeutics, Prince of Wales Hospital, Hong Kong, China

**Keywords:** Asthma, COPD, Overlap, Exacerbations

## Abstract

**Introduction:**

Some patients exhibit characteristics of both asthma and COPD (asthma-COPD overlap [ACO]). There is no gold standard for diagnosing ACO, and limited prospective data exist on the medium-term outcomes of these patients based on the Global Initiative for Asthma (GINA) definition compared to those with asthma or COPD alone. This study assessed the clinical characteristics and risk factors for acute exacerbations (AEs) in ACO patients versus those with COPD or asthma over 3 years.

**Methods:**

Subjects aged over 40 years with a history of asthma and COPD, diagnosed by GINA and Global Obstructive Lung Disease (GOLD) guidelines, were recruited and followed for 3 years. Baseline assessments included a clinical history, symptoms, lung function, a 6-minute walk test (6MWT), blood eosinophil levels, and immunoglobulin E levels. ACO patients were identified per GINA 2017 criteria. AEs were recorded, and risk factors were assessed using Cox regression.

**Results:**

Of 538 patients (299 asthma, 239 COPD), 78 (14.5%) were classified as ACO. Post-bronchodilator forced expiratory volume in 1 s (FEV1) % predicted (mean ± SD) was 83.1 ± 19.0% for asthma, 48.0 ± 19.0% for COPD, and 63.0 ± 22.2% for ACO (*p* < 0.001). Over 3 years, all AEs occurred in 59/272 (21.7%) asthma, 90/188 (47.9%) COPD, and 28/78 (35.9%) ACO patients, while severe AEs requiring hospitalization occurred in 24/272 (8.8%), 79/188 (42.0%), and 20/78 (25.6%), respectively. Compared to asthma patients, ACO patients had a significantly higher risk of first severe AE requiring hospitalization (*p* < 0.0001) but a lower risk compared to COPD patients (*p* = 0.014). Risk factors for AEs (all or severe) were: for asthma, prior AE history, shorter 6MWT distance, and lower lung function; for COPD, prior AE history, lower lung function, higher eosinophil count, and being underweight; for ACO, prior AE history and lower lung function.

**Conclusion:**

In this 3-year prospective study using the GINA definition, ACO patients showed an intermediate risk of severe AEs requiring hospitalization compared to asthma (higher) and COPD (lower), with prior exacerbation history and lower lung function as common risk factors across groups.

**Trial registration:**

gov registration number NCT03272932 (registration date: 9 Jan 2017).

**Supplementary Information:**

The online version contains supplementary material available at 10.1186/s12931-026-03643-0.

## Background

Both chronic obstructive pulmonary disease (COPD) and asthma are prevalent and heterogeneous obstructive airway diseases [1, 2]. A systematic review and modelling study estimated that the global prevalence of COPD was 392 million and 292 million based on the Global Obstructive Lung Disease (GOLD) Criteria of forced expiratory volume in 1 s (FEV1)/forced vital capacity (FVC) ratio of < 70% and lower limit of normal, respectively [3]. On the other hand, the Global Burden of Disease Collaboration in 2019 estimated that asthma affects as many as 262 million people worldwide [4].

Asthma and COPD have distinct characteristics, etiologies, and pathophysiology features; however, patients with asthma or COPD can exhibit concomitant features of both, referred to as asthma-COPD overlap (ACO) [2]. The GOLD and Global Initiative for Asthma (GINA) jointly published the topic ACO for the first time in 2014 [5]. ACO is considered a condition characterised by persistent airflow limitation with features of both asthma and COPD. While ACO is recognised, there is, however, no consensus on its definition.

A systematic review and meta-analysis reported that the prevalence of ACO varied widely among studies from 0.3 to 5.0%, 3.2 to 51.4% and 12.6 to 55.7% in the general population, patients with asthma, and patients with COPD, respectively [6]. There are many definitions of ACO; while some only depend on the doctor’s diagnosis of asthma and COPD, others include a combination of various clinical features of asthma and COPD, different lung function criteria, and CT features and biomarkers, such as eosinophils and fractional exhaled nitric oxide (FeNO) [7]. From the literature, there was scarce information on the prevalence and clinical outcomes of patients with ACO based solely on the GINA definition [8] of ACO, in which ACO was diagnosed taking into consideration features including the age of onset, pattern of symptoms, lung function, lung function between symptoms, past history or family history, time course, chest radiograph features and exacerbations.

Earlier research using different definitions indicated that patients with ACO might face more frequent exacerbations, increased healthcare costs, more hospital admissions, and a higher mortality rate compared to those with only asthma or COPD [9, 10]. A previous study from the United States found that older adults with ACO also had almost 2 and 1.5 times higher expenditures compared to individuals with asthma only and COPD only, respectively [11]. In this study, we evaluate the prevalence of ACO based on the 2017 GINA guideline. We also compared the 3-year exacerbation and mortality rates of ACO subjects against those with asthma or COPD subjects in addition to assessing the risk factors for exacerbations and mortality among ACO, asthma, and COPD patients.

## Methods

This was a 3-year prospective observational study of patients with COPD, asthma, and ACO. The study (ClinicalTrial.gov registration number NCT03272932) was approved by the Joint Chinese University of Hong Kong-New Territories East Cluster Clinical Research Ethics Committee (CREC 2017.331). All recruited subjects had signed informed written consents.

### Subjects

Patients were recruited from the medical outpatient clinics by physicians at the Prince of Wales Hospital in Hong Kong. Patients with a confirmed diagnosis of COPD (clinical features of COPD with post-bronchodilator FEV1/FVC < 0.7 based on the medical records)[1] or asthma (defined as those with a consistent history and prior documented evidence of variable airflow obstruction, with evidence of an increase in FEV1 ≥ 12% and ≥ 200 mL following bronchodilator or bronchial hyperresponsiveness on bronchial provocation testing, when stable) [2] were recruited prospectively. Only subjects aged > 40 years were recruited. The exclusion criteria included patients with a history of other chronic respiratory diseases apart from asthma or COPD, such as bronchiectasis, tuberculosis (TB)-destroyed lung parenchyma, endobronchial TB, and lung cancer, or a history of lung resection. In addition, patients with serious medical diseases like severe congestive heart failure, renal failure or cancer that would significantly affect their survival and follow-up in the subsequent 3 years were excluded. Subjects with COPD and asthma exacerbation could join the study 8 weeks post-recovery from the exacerbation.

### ACO diagnosis

For patients with asthma and COPD recruited, a diagnosis of ACO was based on the presence of the usual features of asthma/COPD (at least 3 for each) with a confirmed diagnosis of COPD or asthma according to the syndromic diagnosis in adults for ACO in the GlNA 2017 guideline [8] There were columns with features suggestive of asthma and COPD, respectively, and if there were similar numbers of checked boxes in each column, the diagnosis of ACO was considered. (Supplementary Table 1). KFW and LR assessed patients to ascertain a diagnosis of asthma, COPD, or ACO. Any disagreements were resolved through discussions or by involving a third person.

### Assessment of the subjects

Subjects were invited to return to our research clinic for assessment. The demographic characteristics of the subjects were collected, including comorbidities, medication use and exacerbations in the previous 12 months. Blood samples were taken for evaluation of blood eosinophil count and total immunoglobulin E (IgE) level. A chest radiograph was performed if not available within the past year.

Spirometry pre- and post-bronchodilator, according to the American Thoracic Society and European Respiratory Society standards, was performed [12]. The updated predicted spirometry values for Hong Kong Chinese were used to calculate the predicted lung function [13]. The Modified Medical Research Council (mMRC) dyspnoea score [14] was assessed in all subjects, while the COPD Assessment Test (CAT) score [15] and Asthma Control Test (ACT) score [16] were administered to subjects with an initial diagnosis of COPD or asthma, respectively. Six-minute walk test [17] was performed on all patients.

The patients were contacted by phone every 6 months for 3 consecutive years to assess exacerbations and mortality. Admissions were cross-checked with clinical records. COPD or ACO exacerbations were referred to as an increase in symptoms requiring treatment with a course of systemic steroids or antibiotics. Asthma exacerbations were referred to as an increase in symptoms requiring treatment with a course of systemic steroids. COPD, asthma or ACO exacerbations requiring hospital admissions were considered as severe exacerbations.

### Statistical analysis

Data analysis was performed using R software (version 4.4.2; R Foundation for Statistical Computing, Vienna, Austria). Patient characteristics were summarised across asthma, COPD, and ACO groups using appropriate descriptive statistics. Chi-square or Fisher’s exact tests were used for categorical variable comparisons, while continuous variables were analysed with either independent t-tests or Mann-Whitney U tests based on their distribution normality. Survival outcomes were evaluated using Kaplan-Meier curves and log-rank tests for group comparisons. Univariate and multivariable Cox regression models were fitted to assess associations between patient factors and clinical outcomes, presenting results as hazard ratios with 95% confidence intervals. All tests were two-sided with significance set at *p* < 0.05. The primary objective of this cross-sectional study was to estimate the prevalence of ACO in patients with asthma or COPD using stratified sampling. Based on an estimated 18% ACO prevalence, 95% confidence level, and 5% absolute precision, 227 subjects were required per stratum. Accounting for a 5% dropout rate, at least 238 subjects were recruited per group (total *n* = 476). The stratified design allows adequate power for separate prevalence estimation within each disease phenotype.

## Results

Subjects were prospectively recruited from January 2018 to December 2020 and then followed up for 3 years from the date of recruitment.

**Fig. 1:**
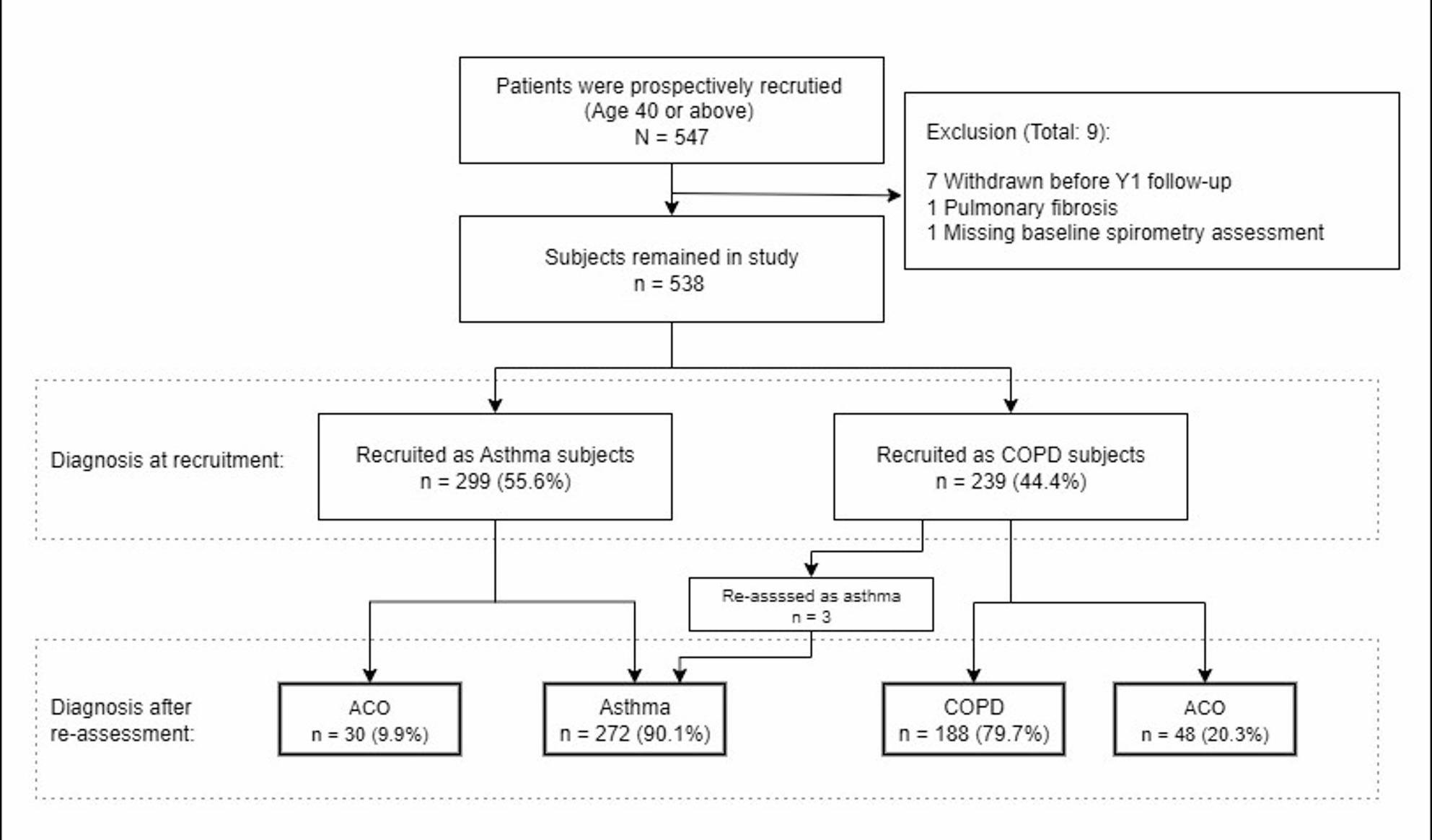
Flow diagram of the establishing the patient cohorts

A total of 547 subjects were recruited, of whom 9 subjects withdrew or were excluded after baseline assessment. Total 538 subjects (299 with an initial diagnosis of asthma and 239 with an initial diagnosis of COPD) were included in the final analysis. Three patients from the COPD group were reclassified as having asthma after initial assessment. Altogether, 78 (14.5%) of the 538 included subjects (30 [10.0%] and 48 [20.1%] from the asthma and COPD diagnosis groups, respectively) were classified as ACO according to the GINA 2017 recommendations. Figure 1 shows the flow diagram of the subjects.

The demographic data of the patients are shown in Table 1. The mean age of the COPD group (73.2 ± 8.1 years) was higher than that of the ACO (67.8 ± 10.6) and asthma (59.2 ± 10.2) groups (*p* < 0.001). There were more males in the ACO compared with the asthma group (90% vs. 35%, *p* < 0.001), and the percentage of males in the ACO and COPD groups was similar. Post bronchodilator FEV1% predicted was higher in asthma when compared to ACO subjects (83.1 ± 19.0 vs. 63.0 ± 22.2, *p* < 0.001) and lower in COPD when compared to ACO subjects (48.0 ± 19.0 vs. 63.0 ± 22.2, *p* < 0.001). COPD patients had more symptoms compared with asthma and ACO patients as measured by the MMRC score. Asthma and ACO patients had similar 6-minute walk test distances, whereas COPD patients had significantly shorter walking distances than the ACO patients (280 [IQR 207–338] vs. 317 [IQR 255–360] m, *p* < 0.001). Regarding comorbidities, more asthma patients had allergic rhinitis compared to COPD and ACO patients. At the same time, hypertension, hyperlipidaemia, diabetes mellitus and ischaemic heart disease were more prevalent in the COPD and ACO groups compared to the asthma group.

**Table 1:** Demographics and clinical characteristics

Characteristics	Asthma *N* = 272	COPD *N* = 188		ACO *N* = 78	*p* (Asthma vs. ACO)		*p* (COPD vs. ACO)
Demographics							
Sex					< 0.001		0.105
Female	177 (65%)	9 (4.8%)		8 (10%)			
Male	95 (35%)	179 (95%)		70 (90%)			
Age (years), mean (SD)	59.2 (10.2)	73.2 (8.1)		67.8 (10.6)	< 0.001		< 0.001
BMI (kg/m^2), mean (SD)	24.7 (4.2)	22.3 (3.5)		23.4 (4.0)	0.014		0.055
BMI (kg/m^2) Group, n (%)					0.008		0.182
Normal (18.5–22.9)	87 (32%)	81 (43%)		25 (32%)			
Underweight (< 18.5)	11 (4.0%)	28 (15%)		11 (14%)			
Overweight ( > = 23)	174 (64%)	79 (42%)		42 (54%)			
Smoking Status, n (%)					**< 0.001**		**0.001**
Non-Smoker	226 (83%)	6 (3.2%)		11 (14%)			
Active Smoker	21 (7.7%)	38 (20%)		22 (28%)			
Ex-Smoker	25 (9.2%)	144 (77%)		45 (58%)			
Severe exacerbations in the past 12 months at baseline., n (%)					**< 0.001**		0.494
0	253 (93%)	128 (68%)		58 (74%)			
1 time	11 (4.0%)	31 (16%)		12 (15%)			
≥2 times	8 (2.9%)	29 (15%)		8 (10%)			
Baseline Assessment Scores							
ACT Score, median (IQR)	20.4 (3.6)	/		/	/		/
CAT Score, median (IQR)	/	9.3 (5.8)		/	/		/
mMRC Score, median (IQR)	0.9 (0.7)	1.6 (0.8)		1.4 (0.7)	**< 0.001**		**0.013**
Baseline Blood Investigations							
WBC Count (x10^9/L), median [Q1, Q3]	6.70 [5.70, 7.85]	7.03 [6.25, 8.40]		6.90 [6.10, 8.40]	0.065		0.501
C-Reactive Protein (mg/L), median [Q1, Q3]	1.4 [0.6, 3.1]	1.6 [0.8, 4.7]		1.8 [0.8, 3.3]	0.149		0.872
Blood Eosinophil (%), median [Q1, Q3]	4.0 [2.0, 6.0]	3.0 [2.0, 5.0]		3.5 [2.0, 5.0]	0.795		0.663
Eosinophil Count (x10^6/L), median [Q1, Q3]	235 [129, 405]	243 [144, 393]		255 [157, 373]	0.768		0.854
Eosinophil Count (x10^6/L) cut-off, n (%)					0.935		0.98
Below 300	166 (61%)	116 (62%)		48 (62%)			
Above or equal to 300	106 (39%)	72 (38%)		30 (38%)			
Total IgE Level (kIU/L), median [Q1, Q3]	182 [52, 348]	78 [24, 229]		98 [42, 467]	0.812		0.014
Total IgE Level (kIU/L), n (%)					0.054		0.151
Below 100	103 (38%)	112 (60%)		39 (50%)			
Above or equal to 100	169 (62%)	76 (40%)		39 (50%)			
Total log (IgE) (kIU/L), median [Q1, Q3]	5.20 [3.94, 5.85]	4.35 [3.16, 5.43]		4.58 [3.74, 6.15]	0.812		0.014
Baseline Spirometry Assessments							
Post-BD FEV1 (L), mean (SD)	1.9 (0.6)	1.1 (0.5)		1.5 (0.6)	< 0.001		**< 0.001**
Post-BD FVC (L), mean (SD)	2.6 (0.8)	2.1 (0.6)		2.6 (0.9)	0.865		**< 0.001**
Post-BD FEV1% predicted, mean (SD)	83.1 (19.0)	48.0 (19.0)		63.0 (22.2)	< 0.001		**< 0.001**
Post-BD FVC % predicted, mean (SD)	90.9 (16.8)	68.5 (18.7)		81.1 (21.2)	< 0.001		**< 0.001**
Post-BD FEV1/FVC ratio (%), mean (SD)	71.4 (11.8)	51.0 (13.5)		57.8 (12.1)	< 0.001		**< 0.001**
Baseline 6-Minute Walking Test							
Distance (m), median [Q1, Q3]	331 [290, 374]	280 [207, 338]		317 [255, 360]	0.050		**< 0.001**
Baseline Medications							
Baseline: Any ICS, n (%)	257 (94.5%)	134 (71.3%)		74 (94.9%)	> 0.999		**< 0.001**
Baseline: Any LABA, n (%)	228 (83.8%)	74 (39.4%)		57 (73.1%)	**0.031**		**< 0.001**
Baseline: Any LAMA, n (%)	84 (30.9%)	73 (38.8%)		40 (51.3%)	**< 0.001**		0.061
Montelukast, n (%)	69 (25%)	4 (2.1%)		12 (15%)	0.065		**< 0.001**
Baseline Comorbidities							
Rhinitis, n (%)	190 (70%)	32 (17%)		25 (32%)	**< 0.001**		**0.007**
Eczema, n (%)	42 (15%)	17 (9.0%)		17 (22%)	0.186		**0.005**
Hypertension, n (%)	90 (33%)	93 (49%)		34 (44%)	0.087		0.382
Hyperlipidaemia, n (%)	75 (28%)	44 (23%)		20 (26%)	0.735		0.698
Diabetes Mellitus, n (%)	43 (16%)	34 (18%)		12 (15%)	0.928		0.596
Ischemic Heart Disease, n (%)	10 (3.7%)	20 (11%)		8 (10%)	**0.036**		0.926
Heart Failure, n (%)	1 (0.4%)	6 (3.2%)	4 (5.1%)		**0.01**		0.486

*ACT* Asthma Control Test, *CAT *COPD Assessment test *IgE *Immunoglobulin E, *ICS *inhaled corticosteroid, *IQR* interquartile range, *LABA *long-acting beta-agonist, *LAMA *long-acting muscarinic antagonist

**Fig. 2:**
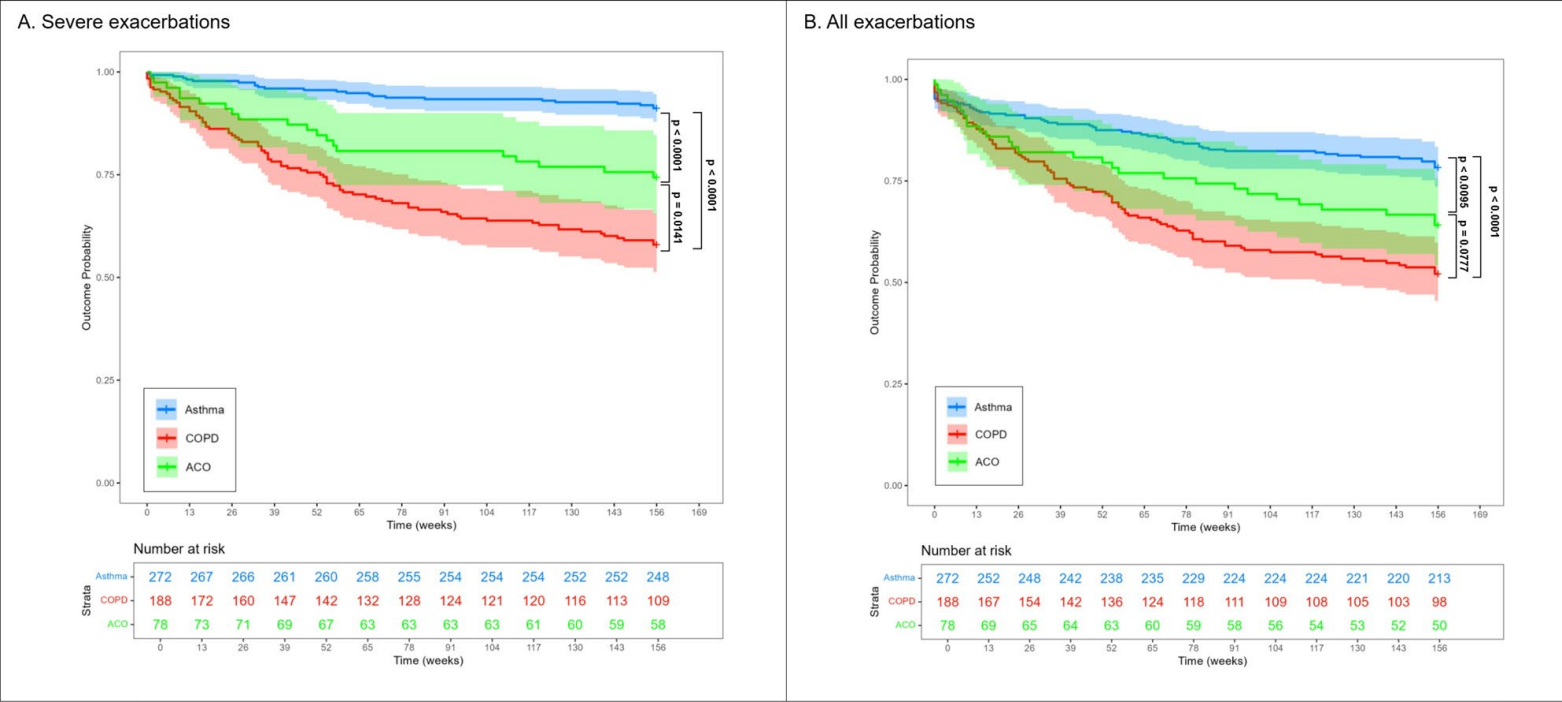
Kaplan-meier curve for cumulative incidence of exacerbations over 3 years. *p*-values by long rank tests. **A** Severe acute exacerbations; **B** All acute exacerbations

Among the asthma, COPD, and ACO subjects, 59/272 (22%), 90/188 (48%) and 28/78 (36%) had exacerbations (all) in the following 3 years after baseline assessments. Severe exacerbations occurred in 24/272 (8.8%), 79/188 (42%), and 20/78 (26%) of the asthma, COPD, and ACO patients, respectively, over 3 years. Subjects in the ACO group had a significantly higher risk of experiencing their first severe exacerbation requiring hospitalisation compared to those in the asthma group (*p* < 0.0001). Patients in the COPD group, in contrast, had a significantly higher risk of encountering the first severe exacerbation compared to those of the ACO group (*p* = 0.014) (Fig. 2a). Similarly, the ACO group had a significantly higher incidence of exacerbation (all) over the 3-year follow-up period compared to the asthma group (*p* = 0.01), but no significant difference was observed between the COPD and ACO group (*p* = 0.08) (Fig. 2b).

**Fig. 3:**
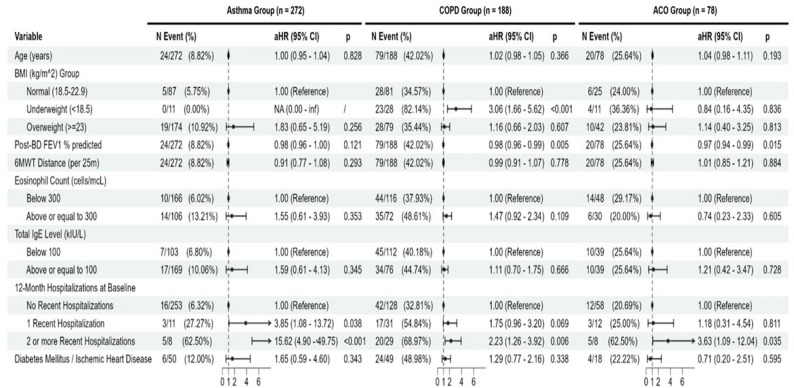
Cox regression (multivariate) for risk factors of first severe acute exacerbations for patients with asthma, COPD and asthma-COPD overlap. 6MWT = 6 min Walk test, IgE=Immunoglobulin E

**Fig. 4:**
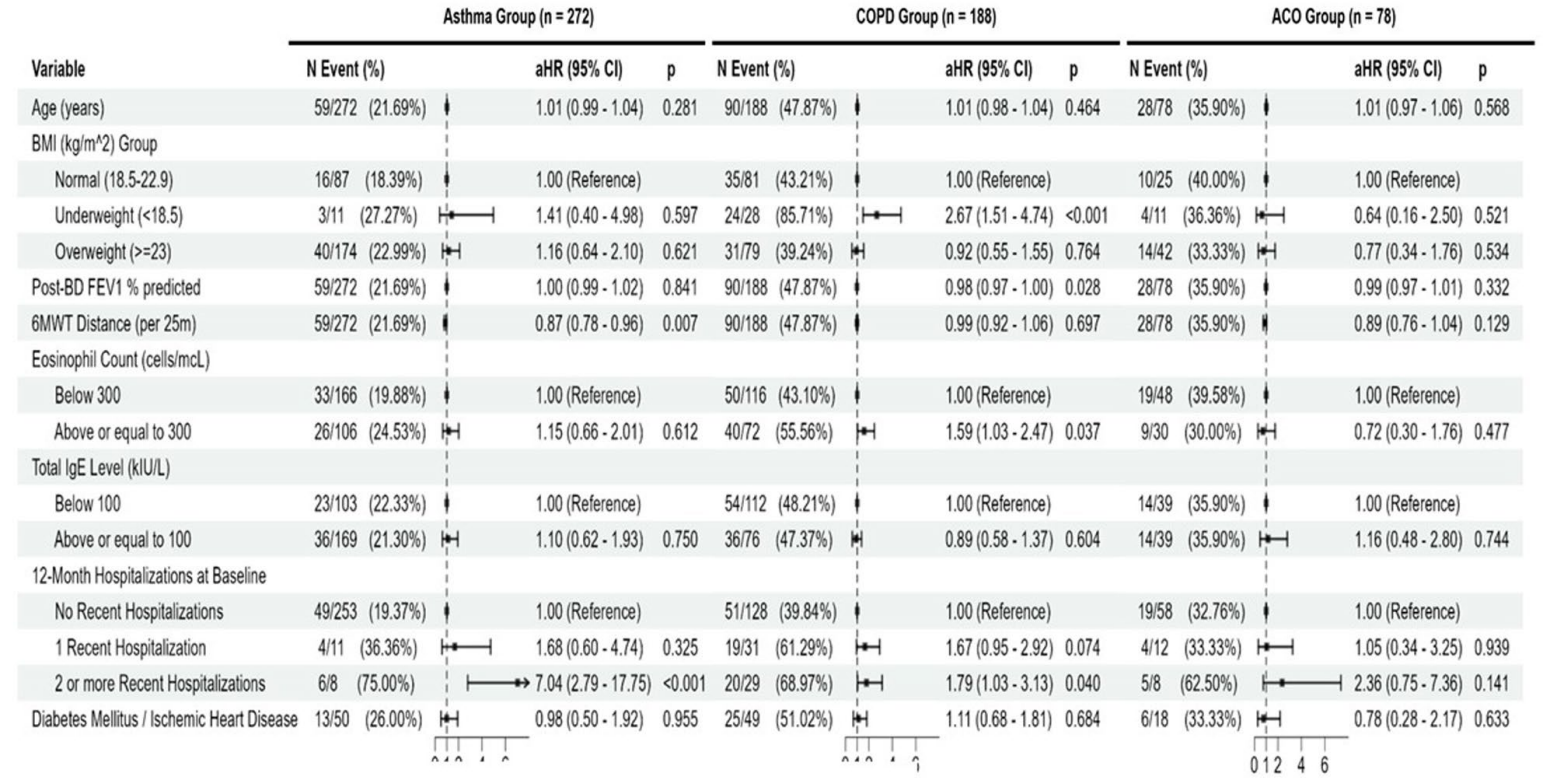
Cox regression (multivariate) for risk factors of first exacerbations (all) for patients with asthma, COPD and asthma-COPD overlap.6MWT = 6 min Walk test, IgE=Immunoglobulin E

For first severe exacerbations requiring hospitalizations, multivariate Cox regression results showed that past 1-year severe exacerbation history at baseline was a statistically significant risk factor in the asthma group for either 1 hospitalization with adjusted hazard ratio (aHR) 3.85 (95% CI: 1.08 − 13.72, *p* = 0.038) and 2 or more hospitalizations with aHR 15.62 (95% CI: 4.90-49.75, *p* < 0.001) (Fig. 3). For the COPD group, significant risk factors for incidence of severe exacerbation included being underweight with (Body mass index [BMI] < 18.5 kg/m2 at baseline with aHR of 3.06 (95% CI 1.66–5.62, *p* < 0.001) with reference to patients within normal BMI range, lower post-bronchodilator FEV1% predicted with aHR of 0.98 (95% CI: 0.96–0.99, *p* = 0.005) (per percentage change), and history of severe exacerbation in the past year where 2 or more exacerbations had aHR of 2.23 (95% CI: 1.26–3.92, *p* = 0.006). For the ACO group, both post-BD FEV1% predicted (aHR 0.97; 95% CI: 0.94–0.99; *p* = 0.015) and 2 or more severe exacerbations in the past year at baseline (aHR 3.63; 95% CI 1.09–12.04; *p* = 0.035) were statistically significant risk factors, Similar pattern in risk profile is also observed in the incidence of all exacerbations across the asthma COPD and ACO groups, except where 6-minute walking distance at baseline associated with greater all exacerbation incidence risk in the asthma group with aHR 0.87 (95%CI: 0.78–0.96; *p* = 0.007) and greater baseline eosinophil level ( > = 300 cells/mcL) significantly associates with greater risk with aHR of 1.59 (95% CI: 1.03–2.47, *p* = 0.037) in the COPD group. The relationship between eosinophil count (unadjusted for other risk factors) and exacerbations for COPD and ACO groups was presented in Supplementary Table 2. No individual statistically significant risk factors were identified in the ACO group for all exacerbation incidence. (Fig. 4).

**Fig. 5:**
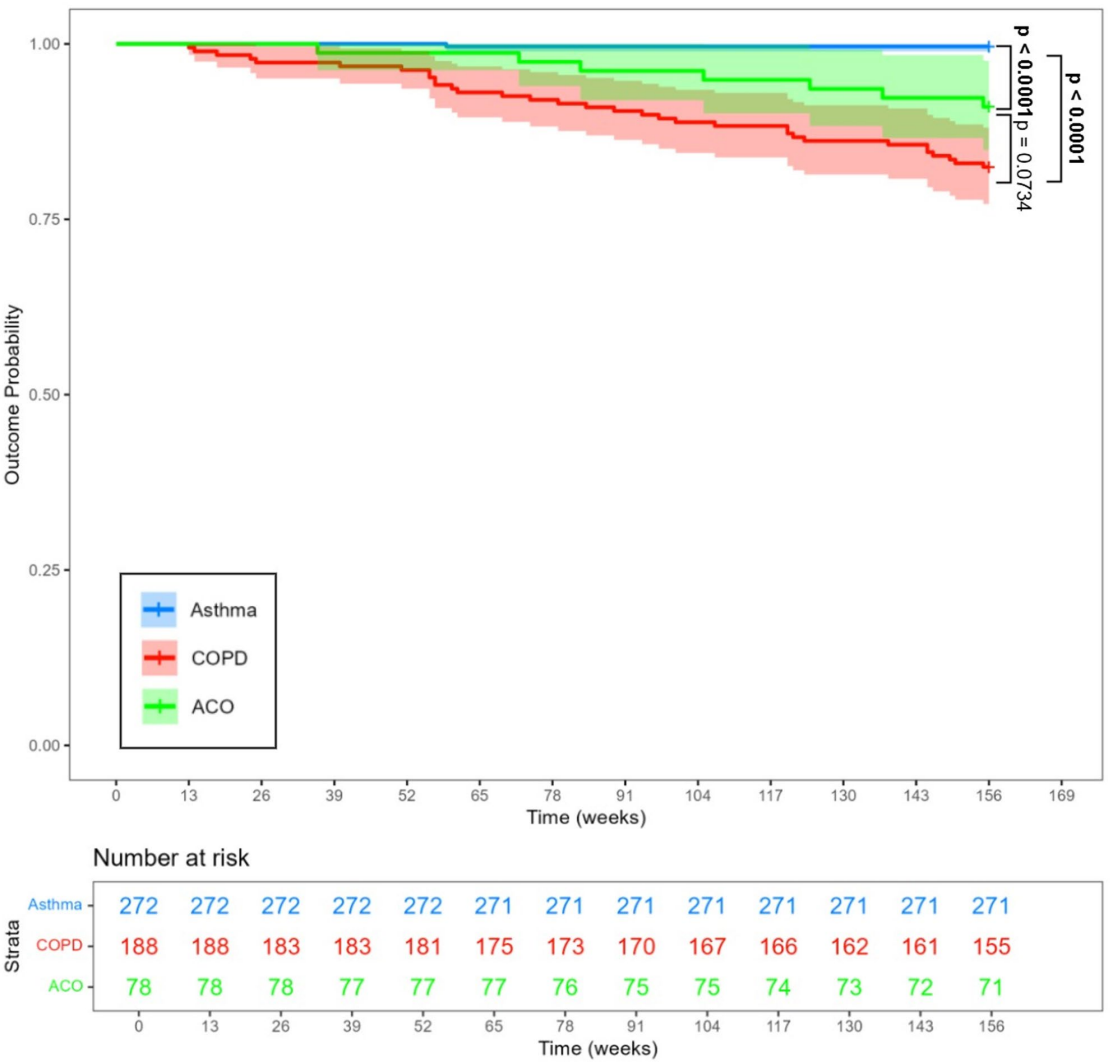
Kaplan-meier curve for cumulative incidence of mortality over 3 years. *p*-values by long rank tests

The 3-year all-cause mortality rates for asthma, COPD, and ACO patients were 1/272 (0.4%), 33/188 (17.6%), and 7/78 (9.0%), respectively. The Kaplan-Meier curve for mortality is shown in Fig. 5. The mortality of ACO patients was much higher when compared to asthma subjects (*p* = 0.0001), but the mortality of COPD patients was statistically not significantly higher than ACO subjects (*p* = 0.074). Risk factors for mortality in 3 years for COPD included older age, BMI indicating underweight, shorter 6-minute walk distance, and exacerbations in the past year. No independent statistically significant risk factors were identified for the mortality of ACO patients. The results for COPD patients are summarised in Supplementary Fig. 1. Asthma and ACO mortality were too low to allow for statistical analysis.

## Discussion

This prospective study has found that the prevalence of ACO among patients with an initial diagnosis of asthma and COPD, using the GINA 2017 criteria, was 14.5%. This prospective study had the strength of a 3-year follow-up that allowed longitudinal assessments of patients. ACO compared to asthma, and COPD compared with ACO patients, had a significantly higher risk of first severe exacerbations requiring hospitalisations over a period of 3 years. The mortality of ACO patients were much higher when compared to asthma, but the mortality of COPD patients was not significantly higher than that of ACO subjects.

The definition of ACO varies across different studies [18, 19]. To our knowledge, few prospective studies have utilised the GINA definition to evaluate the prevalence of ACO patients [20, 21]. Additionally, there is limited data on the longitudinal follow-up of ACO patients using GINA criteria. A prospective study from Korea which assessed the prevalence of ACO based on GINA criteria, with a follow-up period of at least one year, found that 46.2% of 301 COPD patients met the GINA definition for ACO, a figure that exceeds the prevalence observed in our current study. Notably, patients with an initial diagnosis of asthma were not included in this research. Furthermore, comparisons of exacerbation rates between COPD and asthma patients were not conducted, and mortality data were not reported [20]. Some studies defined ACO based on physician or patient-reported diagnoses of concomitant asthma or COPD without considering lung function parameters [22, 23]. Certain guidelines, such as the Spanish guideline, employ specific combinations of major and minor clinical criteria (e.g., persistent airflow limitation, smoking history, documented history of asthma, history of atopy or allergic rhinitis, bronchodilator response, and elevated peripheral blood eosinophils) for diagnosing ACO [24, 25]. Some guidelines even incorporate FeNO and CT imaging parameters into their definitions of ACO [26]. Overall, the prevalence of ACO among asthma and COPD patients in this study aligns with findings from previous studies based on different definitions [6, 18]. However, the methodologies of these studies varied, making direct comparisons challenging. Given the lack of a universally accepted definition, it is crucial to conduct thorough assessments of ACO based on specific criteria to provide more comprehensive information for the management of patients with ACO.

Regarding the terminology of ACO, since the 2017 GINA guideline, [8]. GINA no longer recommends the term “ACO Syndrome”, as this was often interpreted as implying a single disease. The latest 2025 GINA guideline [2] stated that ACO is a descriptor for patients often seen in clinical practice, who comprise a heterogeneous group and do not refer to a single disease entity. To avoid confusion, the term “asthma+COPD” is now preferred. As this study was planned in 2017 and commenced in 2018, the term ACO was used because it was the prevailing terminology at the time. Variable airflow limitation is necessary for an asthma diagnosis, and the inclusion criteria for asthma patients in this study included bronchodilator reversibility. This definition did not exclude COPD patients. In the UPLIFT study, [27, 28] 52% of COPD patients met the criteria for bronchodilator reversibility, with ≥ 12% and ≥ 200 mL. With this inclusion of asthma patients, the investigators assessed individuals based on multiple parameters (as shown in Supplementary Table 1), allowing for appropriate classification of patients as having asthma, COPD, or ACO for comparison.

Previous research on ACO patients has identified several key characteristics [29] Male gender, smoking exposure, reduced lung function as measured by spirometry, and diminished physical performance are more closely associated with COPD and ACO compared to asthma. However, individuals with ACO tend to be younger, more frequently female, have a higher BMI, lower lifetime smoking exposure, and experience more symptoms, lower quality of life and exacerbations than those with COPD [29, 10, 30]. The prevalence of comorbidities differs across asthma, COPD, and ACO [31, 29]. Asthma patients often have associated conditions such as allergic rhinitis, conjunctivitis, atopic skin disorders, and obesity. In contrast, COPD is typically linked to multiple comorbidities, with common conditions including cardiovascular disease, diabetes, sarcopenia, and depression. ACO patients exhibit comorbidities characteristic of both asthma and COPD. Our findings align with these observations regarding ACO patient characteristics.

Inhaled corticosteroid (ICS)-based therapy has been recommended by GINA for asthma patients to improve symptom control and reduce the risk of exacerbations [2]. For COPD, bronchodilators are the mainstay of treatment, and ICS would be beneficial for patients with a history of exacerbations and high eosinophil count [1] Triple therapy with an ICS, long-acting beta-agonist (LABA), and long-acting antimuscarinic agent (LAMA) was not yet available at the start of this study. Despite 94% of our asthma subjects being treated with ICS, the rate of exacerbations remained high, with 22% experiencing any exacerbation and 8.8% suffering from severe exacerbations over three years. A previous study analysing data from over 200,000 asthma subjects each in the United States and the United Kingdom reported that 12.5% and 8.4%, respectively, experienced at least one exacerbation during a 12-month follow-up period. Furthermore, it found that prior exacerbations increased the risk of future exacerbations [32]. In our asthma cohort, approximately 7% had experienced any asthma exacerbations in the past 12 months at baseline. Although most of the asthma patients were reported to be on ICS, with some on the maintenance and reliever therapy (MART) strategy, we did not assess adherence to ICS in this study, which may have contributed to the high rate of exacerbations.

A systematic review involving eleven studies indicated that patients with ACO had significantly more exacerbations than those with asthma or COPD [33]. An epidemiological study in the US found that ACO patients had increased rates of hospitalizations and emergency department visits compared to those with COPD [34]. A study from Copenhagen reported that exacerbations requiring hospital admission were notably higher among patients with asthma, COPD, early-onset ACO, and late-onset ACO, compared with control subjects, with hazard ratios of 14.7, 23.8, 39.5, and 83.5, respectively [35]. Conversely, a Japanese study indicated that ACO patients receiving appropriate treatment did not exhibit poor clinical outcomes compared with COPD patients [36]. Our findings align with prior research, confirming that ACO patients experience more severe exacerbations than those with asthma. However, our ACO patients exhibited a lower risk of severe exacerbations compared to COPD patients. This is likely attributable to the fact that 95% of our ACO patients were on ICS-based therapy, consistent with the Japanese study [36] that found no increased exacerbation risk in appropriately treated ACO patients compared to those with COPD.

Variability in exacerbation rates and risk factors for asthma, COPD, and ACO across studies may stem from differences in patient severity and definitions of ACO [7]. For asthma patients, factors such as elevated blood eosinophils, increased FeNO levels, history of attacks, disease severity, reduced lung function, and symptom frequency have been linked to subsequent exacerbations [37]. Our observation of lower lung function and a history of exacerbations in the previous year aligns with these findings. An earlier study found that in severe asthma, symptoms and BMI were correlated with exercise capacity as measured by the 6-minute walk test [38]. In our study, a shorter 6-minute walk distance emerged as a risk factor for asthma exacerbations, even when adjusted for age, BMI, lung function, previous exacerbation history, and comorbidities. This shorter distance may indicate more severe symptoms and contribute to a higher incidence of exacerbations.

We also found that being underweight and having a higher blood eosinophil count were associated with COPD exacerbations, consistent with previous research. A recent systematic review indicated that underweight patients, but not those who are overweight or obese, have an increased risk of COPD exacerbation compared to individuals with a normal BMI [39]. The relationship between blood eosinophils and future exacerbations in COPD remains contentious. Our study showed that higher blood eosinophil counts were linked to an increased risk of all acute exacerbations of COPD, but not for severe exacerbations requiring hospitalization. Previous studies on eosinophil levels at admission have yielded mixed results, with some finding no association with future exacerbation risks while others identified significant links [40–43]. Some studies have suggested that eosinophil levels correlate with acute exacerbation of COPD in real-world and primary care settings [42, 43]. Eosinophil counts appear to predict all exacerbations, though not specifically severe ones. The high rate of ICS use among participants in this study might also influence the exacerbation rates. For ACO patients, we identified that lower lung function and a history of previous exacerbations were independent factors associated with a higher risk of severe exacerbations, but not all exacerbations. The heterogeneity of ACO patients suggests that a larger sample size may be necessary for more comprehensive risk factor assessments.

This study found that COPD and ACO patients had a significantly higher mortality risk than asthma patients. COPD showed a trend toward higher mortality than ACO, though not statistically significant. Asthma mortality was low (0.4%) in this study, likely due to the younger patient age, fewer cardiovascular comorbidities, most being non-smokers and high usage of ICS. These findings align with an 18-year Finnish national health survey, which also showed higher mortality in COPD and ACO compared to asthma, with no significant mortality difference between ACO and COPD [29]. This is expected if asthma is treated appropriately with ICS. A Copenhagen study reported elevated respiratory and all-cause mortality in asthma, COPD, and ACO patients compared to never-smokers without airway disease [35]. As our study lacks a control group without airway disease, such comparisons were not possible. In this study, we found that lower BMI was associated with higher mortality in the COPD patients, and this concurs with previous reports on the “obesity paradox’ of COPD, with obese patients having lower mortality [44]. Since mortality was not the primary focus of our study, longer-term research with larger sample sizes is needed to better understand the risk factors for mortality in our patients.

One limitation of this study is that it was a single-centre study which aimed to identify patients with ACO using the 2017 GINA guidelines, without comparing other definitions. Additional tests, such as FeNO, CT thorax, or sputum eosinophil counts, were not performed, but these could have provided further insights into emphysema or type 2 inflammation. Furthermore, our assessment only considered the effects of smoking and did not take into account other environmental factors, such as ambient air pollution or occupational exposures, that may influence the development of both asthma and COPD [45, 46]. However, the study had a prospective design with a 3-year follow-up, incorporating lung function tests and the 6-minute walk test, and these are often not available in database studies. Additionally, it utilised the 2017 GINA classification, a definition not commonly used in prior studies due to its recent introduction.

## Conclusions

This prospective study has found a prevalence of 14.5% for ACO among those initially diagnosed with asthma or COPD, based on the GINA criteria. Over three years, ACO patients faced a higher risk of severe exacerbations compared to asthma patients but a lower risk compared to COPD patients. Lower lung function and a history of exacerbations were associated with severe exacerbations requiring hospitalization in ACO patients. Further understanding of ACO patient characteristics and risks is crucial for developing personalized management strategies for this heterogeneous group.

(Word count 3879)

## Supplementary Information

Supplementary Material 1.

## Data Availability

The datasets used and/or analysed during the current study are available from the corresponding author on reasonable request.

## Abbreviations

ACO: Asthma-COPD overlap
ACT: Asthma Control Test
aHR: adjusted hazard ratio
BMI: Body mass index
CAT: COPD Assessment Test
COPD: Chronic obstructive pulmonary disease
FeNO: Fractional exhaled nitric oxide
FEV1: Forced expiratory volume in 1 s
FVC: Forced vital capacity
GINA: Global Initiative for Asthma
GOLD: Global Obstructive Lung Disease
ICS: Inhaled corticosteroid
IgE: Immunoglobulin E
mMRC: Modified Medical Research Council
TB: Tuberculosis
