# Supplementary material for: A 3-year prospective study to assess clinical characteristics and risk factors for exacerbations in patients with asthma-COPD overlap based on the GINA guideline compared with patients with asthma and COPD
Source: Respir Res. 2026 Mar 29;27:202. doi: 10.1186/s12931-026-03643-0 (PMC13151370; doi:10.1186/s12931-026-03643-0)
Supplement: Supplementary file 1 — Supplementary Material 1. [file 12931_2026_3643_MOESM1_ESM.docx]

Supplementary Table 1: Global Initiative Asthma Asthma-COPD Overlap diagnostic recommendation (2017)


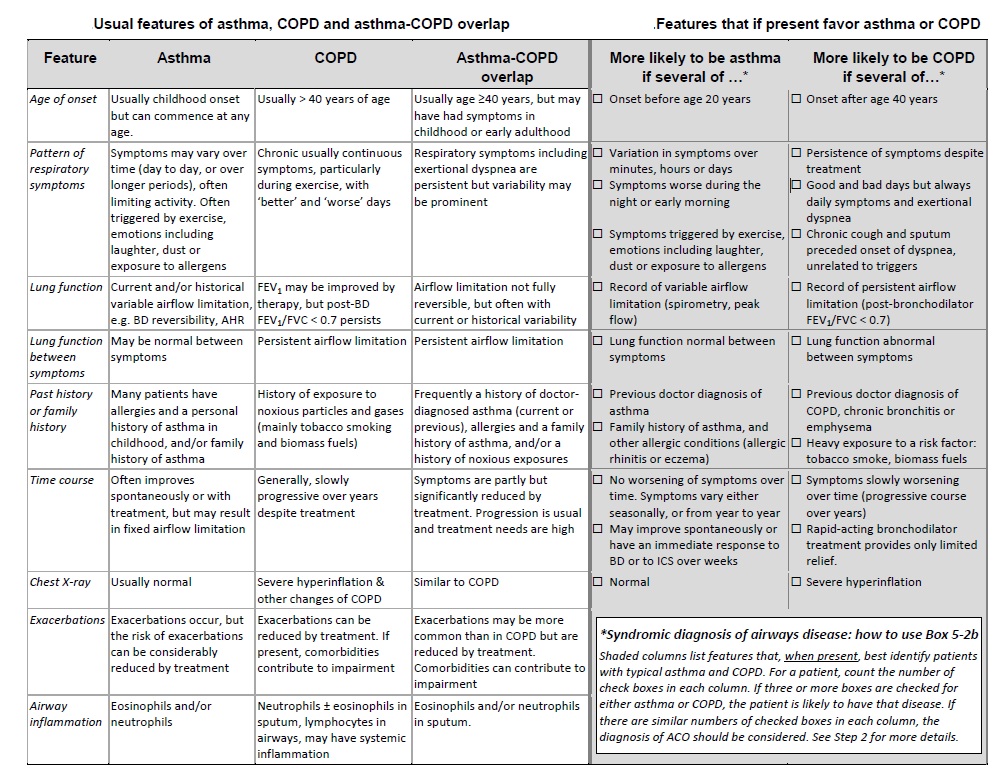


Source: Global Strategy for Asthma Management and Prevention 2017 (obtained approval from Global Initiative of Asthma to use this figure)

Supplementary Table 2. Relationship between eosinophil count (unadjusted for other risk factors) and exacerbations for COPD and ACO groups

|  | **COPD** | **ACO** | **P value** |
| --- | --- | --- | --- |
| Blood eosinophils <300 x10^6/L | | | |
| Any first exacerbations |  |  | 0.7318 |
| Without exacerbations | 65 (56.0%) | 29 (60.4%) |  |
| With exacerbations | 51 (44.0%) | 19 (39.6%) |  |
|  |  |  |  |
| Any first severe exacerbations |  |  | 0.3222 |
| Without exacerbations | 71 (61.2%) | 34 (70.8%) |  |
| With exacerbations | 45 (38.8%) | 14 (29.2%) |  |
|  | | | |
| Blood eosinophils ≥300 x10^6/L | | | |
| Any first exacerbations |  |  | 0.0444 |
| Without exacerbations | 33 (45.8%) | 21 (70.0%) |  |
| With exacerbations | 39 (54.2%) | 9 (30.0%) |  |
|  |  |  |  |
| Any first severe exacerbations |  |  | 0.0191 |
| Without exacerbations | 38 (52.8%) | 24 (80.0%) |  |
| With exacerbations | 34 (47.2%) | 6 (20.0%) |  |

Data are presented as the number of patients (%)


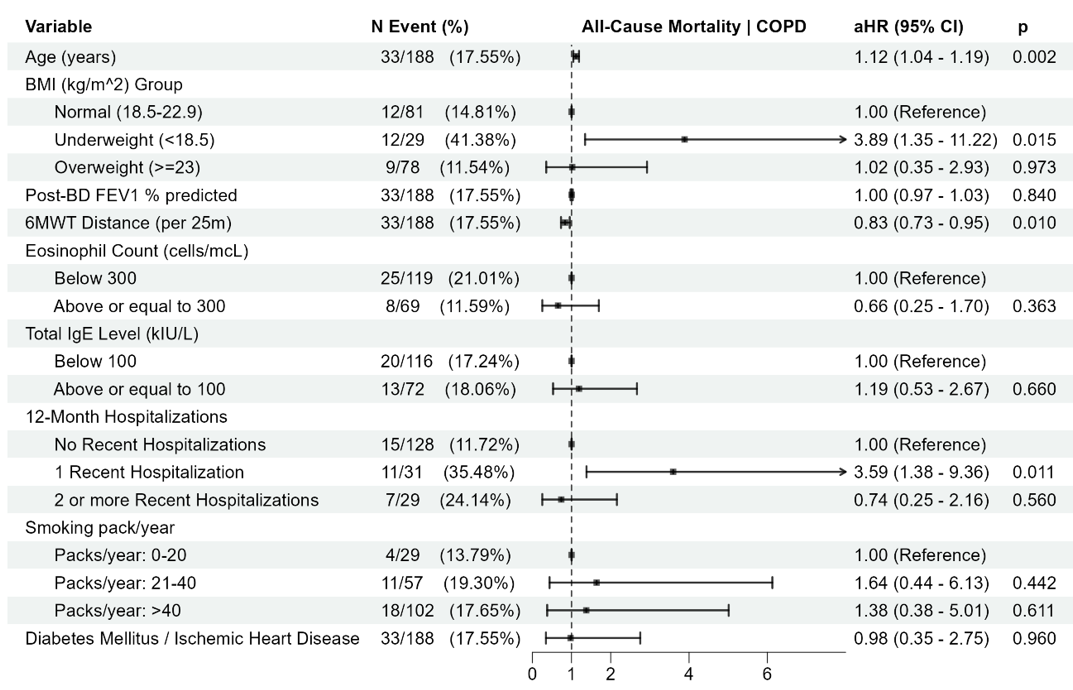


Supplementary Figure 1. Cox Regression (multivariate) for risk factors of mortality for patients with COPD

6MWT=6 Minute Walk test, IgE=Immunoglobulin.
